# Influence of the Draining Lymph Nodes and Organized Lymphoid Tissue Microarchitecture on Susceptibility to Intradermal *Trypanosoma brucei* Infection

**DOI:** 10.3389/fimmu.2020.01118

**Published:** 2020-06-03

**Authors:** Omar A. Alfituri, Barry M. Bradford, Edith Paxton, Liam J. Morrison, Neil A. Mabbott

**Affiliations:** The Roslin Institute and Royal (Dick) School of Veterinary Sciences, University of Edinburgh, Edinburgh, United Kingdom

**Keywords:** *Trypanosoma brucei*, trypanosomes, spleen, lymph nodes, B cells, lymphotoxin

## Abstract

Infection of the mammalian host with African trypanosomes begins when the tsetse fly vector injects the parasites into the skin dermis during blood feeding. After injection into the skin, trypanosomes first accumulate in the draining lymph node before disseminating systemically. Whether this early accumulation within the draining lymph node is important for the trypanosomes to establish infection was not known. Lymphotoxin-β-deficient mice (LTβ^−/−^ mice) lack most secondary lymphoid tissues, but retain the spleen and mesenteric lymph nodes. These mice were used to test the hypothesis that the establishment of infection after intradermal (ID) *T. brucei* infection would be impeded in the absence of the skin draining lymph nodes. However, LTβ^−/−^ mice revealed greater susceptibility to ID *T. brucei* infection than wild-type mice, indicating that the early accumulation of the trypanosomes in the draining lymph nodes was not essential to establish systemic infection. Although LTβ^−/−^ mice were able to control the first parasitemia wave as effectively as wild-type mice, they were unable to control subsequent parasitemia waves. LTβ^−/−^ mice also lack organized B cell follicles and germinal centers within their remaining secondary lymphoid tissues. As a consequence, LTβ^−/−^ mice have impaired immunoglobulin (Ig) isotype class-switching responses. When the disturbed microarchitecture of the B cell follicles in the spleens of LTβ^−/−^ mice was restored by reconstitution with wild-type bone marrow, their susceptibility to ID *T. brucei* infection was similar to that of wild-type control mice. This effect coincided with the ability to produce significant serum levels of Ig isotype class-switched parasite-specific antibodies. Thus, our data suggest that organized splenic microarchitecture and the production of parasite-specific Ig isotype class-switched antibodies are essential for the control of ID African trypanosome infections.

## Introduction

African trypanosomes are single-celled protozoan parasites that are transmitted between mammalian host species via blood-feeding tsetse flies of the genus *Glossina*. The *Trypanosoma brucei rhodesiense* and *T. b. gambiense* subspecies cause human African trypanosomiasis in endemic regions of sub-Saharan Africa. Animal African trypanosomiasis is caused by *Trypanosoma congolense, Trypanosoma vivax* and *T. brucei*, and inflicts substantial economic strains on the African livestock industry. These parasites exist entirely extracellularly within their hosts, and the life cycle in the mammalian host is initiated by the intradermal (ID) injection of metacyclic trypomastigotes into the skin dermis by the tsetse fly vector. After ID injection the parasites can directly infect the lymphatics (1) before disseminating to the draining lymph nodes and then systemically via the bloodstream (2–5). Soon after ID injection the parasites also undergo morphological change into the long-slender bloodstream form stages that are specifically adapted for survival within the hostile environment of the mammalian bloodstream.

Although much attention has been given to the study of experimental trypanosome infections initiated by intraperitoneal (IP) or intravenous injection, comparatively little is known of the host-specific factors that are important for the effective control of ID transmitted trypanosome infections. However, Wei and colleagues have shown that infection of mice by the IP route establishes a detectable parasitemia earlier than after ID injection, with a higher parasite burden (6). Furthermore, the same study suggested mice were ~100X less susceptible to ID injected *T. brucei* infection when compared to mice infected by the IP route.

Lymphotoxin-α (LTα) and lymphotoxin-β (LTβ) are members of the TNF superfamily and form a heterotrimer (LTα_1_β_2_) to allow signaling through the LTβ receptor (LTβR) (7). Signaling via LTβR during embryonic development is essential for the development of the peripheral lymph nodes (8). Subsequently, post-natal lymphocyte-derived LTα_1_β_2_-mediated stimulation is also essential for the maintenance of organized tissue microarchitecture within the secondary lymphoid organs. As a consequence, mice deficient in LTα, LTβ, or LTβR lack most peripheral lymph nodes and have grossly disorganized microarchitecture in their remaining secondary lymphoid organs such as the spleen (8–15). For example, these mice have disturbed B cell follicles, lack germinal centers (GC) and as a consequence have a significantly reduced ability to produce high affinity antigen-specific class-switched immunoglobulins (Ig) upon immunization. Therefore, in this study we used LTβ^−/−^ mice that lack most peripheral lymph nodes to determine the requirement of the draining lymph nodes for the effective establishment of infection after intradermal (ID) inject with *T. brucei*. Using these mice we show that the early accumulation of the trypanosomes in the draining lymph nodes was not essential to establish systemic infection. Although LTβ^−/−^ mice were able to control the first parasitemia wave as effectively as wild-type mice, they were unable to control subsequent parasitemia waves. Subsequent experiments suggested that the inability of LTβ^−/−^ mice to control the subsequent parasitemia waves coincided with the lack of organized B cell follicles in their spleens, and their impaired ability to mount parasite-specific Ig isotype class-switched antibody responses. This study provides important insight into the important host factors that are essential for the efficient control of ID trypanosome infections.

## Materials and Methods

### Mice

Six to 8 weeks old female C57BL/6J mice (Charles Rivers, Harlow, England) and lymphotoxin-β-deficient (LTβ^−/−^) mice (15) were used throughout this study. Mice were maintained in individually ventilated cages and provided food and water *ad libitum*. All procedures using experimental mice were carried out under the authority of the appropriate project and personal licenses, in accordance with the United Kingdom Home Office regulations and the Animals (Scientific Procedures) Act 1986. Ethical approvals were obtained from The Roslin Institute's and University of Edinburgh's ethics committees.

### γ-Irradiation and Reconstitution With Donor Bone Marrow

Where indicated, recipient female LTβ^−/−^ mice (~20 g each, 6–8 weeks old) were γ-irradiated twice (5 Grays each) at a 4 h interval. Bone marrow from the long bones of donor mice was aseptically prepared as a single cell suspension at ~1 × 10^7^ cells/ml in HBSS (Life Technologies, Paisley, UK). Approximately 18 h after the irradiation the recipient mice each received 100 μl of fresh donor bone marrow by intravenous injection into the tail vein. Recipient mice were allowed to recover for 10 weeks prior to their use in subsequent experiments.

### Trypanosomes

The pleomorphic wild-type *T. b. brucei* strain STIB247 was used throughout this study. These trypanosomes were originally isolated from a hartebeeste (*Alcelaphus buselaphus*) in Tanzania's Serengeti National Park in 1971 (16). The trypanosomes were axenically cultivated *in vitro* as described previously (1). Prior to their use in the *in vivo* studies described, ~1x10^5^ axenically cultivated trypanosomes were first injected IP into groups of C57BL/6J mice. Blood was collected at the peak of the first parasitemia and used as a fresh source of *in vivo*-adapted parasites for each experiment. In the experiments described the mice were infected ID with ~1 × 10^2^ or 1 × 10^5^ *T. b. brucei* STIB247 parasites, where indicated.

### Comparison of Serum Immunoglobulin (Ig) Isotype Levels by ELISA

Serum IgM and IgG isotype levels were measured by ELISA. For total Ig measurements a capture ELISA was used whereby 96 well plates (Immulon 4HBX 96-Well Micro Plate, SLS, UK) were first coated with 50 μl of either purified rat anti-mouse IgM coating antibody (BD 553435 (II/41), BD Biosciences, USA) or purified polyclonal goat anti-mouse coating Ig antibody (BD 553998, BD Biosciences, USA), each prepared at 5 μg/ml in p-nitrophenyl phosphate substrate buffer. The plates were sealed and incubated overnight at 4°C. The plates were then blocked using 100 μl of 1% bovine serum albumin (BSA)/PBS (Sigma-Aldrich) to each well and incubated at 37°C for 1 h, and then washed 5 times in 0.05% Tween/PBS. Sera (50 μl/well) was then added in 0.1% BSA/PBS and incubated for 1 h at 37°C at the following dilutions: IgM, 1:400; IgG1 1:800; IgG2c, 1:50; IgG3, 1:800. The serum dilutions used were based on previously established titrations. Serial dilutions of normal mouse IgM (clone RMM-1), normal mouse IgG1 (clone RMG1-1), normal mouse IgG2c (clone RMG2a62) or normal mouse IgG3 (clone R40-82) (all from Biolegend, USA) were used to establish standard curves. After washing, 50 μl of Ig-subclass-specific biotinylated secondary antibodies (anti-IgM, clone G155-228, BD Biosciences; anti-IgG1, clone MG1-45, BioLegend; anti-IgG2c, clone MG2a-53, BioLegend; anti-IgG3, clone MG3-25, clone MG3-25) were applied in 1% skimmed milk/0.1% BSA/PBS (1/500 dilution), and incubated for 1 h at 37°C. Plates were subsequently washed and 50 μl streptavidin-conjugated horseradish peroxidase was added (1/1,000 dilution) and incubated at 37°C for 1 h. After a final wash, bound peroxidase activity was revealed by adding 50 μl of SureBlue TMB microwell Peroxidase Substrate to each well (KPL, SeraCare 5120-0075, Massachusetts, USA), and the reaction stopped using HCl (1M). Optical density (OD) was then determined using a Perkin Elmer Wallac 1420 Victor^2^ Microplate Reader (GMI, USA) at 450 nm with 620 nm used as the reference OD value.

To estimate trypanosome-specific Ig levels an indirect ELISA was used whereby the plates were first coated with 50 μl of trypanosome lysate (0.7 μg protein/ml) in 0.1 M bicarbonate buffer, in place of the purified rat anti-mouse IgM purified polyclonal goat anti-mouse coating Ig antibodies. The remainder of the assay was repeated as above. A potential limitation of this approach being that the binding characteristics of the coating Ig antibodies used in the capture ELISA may differ from that of the trypanosome lysate used in the indirect assay.

### Immunohistochemistry

Spleens were snap frozen at the temperature of liquid nitrogen, and 10 μm thick sections cut using a cryostat. Sections were then fixed in acetone and follicular dendritic cells detected using mAb 8C12 to detect CD35 (BD Biosciences, Oxford, UK) and B cells detected using rat anti-mouse B220 mAb (clone RA3-6B2, Life Technologies). Sections were subsequently stained with species-specific secondary antibodies conjugated to Alexa Fluor 488 (green) or Alexa Fluor 594 (red) dyes (Life Technologies). Sections were then imaged using a Zeiss LSM710 confocal microscope (Zeiss, Welwyn Garden City, UK).

### Statistics

ELISA data were tested for normal distribution using Shapiro-Wilk and Kolmogorov Smirnov tests. Data that passed these normality tests were subsequently compared using a Student's *t*-test. Data that did not pass these tests were compared using a Mann-Whitney *U* test. These analyses were performed using GraphPad Prism v.8.0 (GraphPad Software Inc. San Diego, USA). To compare the parasitemias between different groups, linear mixed effects models were performed using RStudio. These were used to statistically compare the quadratic (squared) and cubic curve effect of the infected mouse parasitemia across the observation period. Mean peak parasitemias were compared using a Student's *t*-test or Tukey's multiple comparisons test. *P* < 0.05 were accepted as significant.

## Results

### LTβ^-/-^ Mice Lack Most Secondary Lymphoid Tissues

First, LTβ^−/−^ mice and C57BL/6J wild-type (WT) control mice were injected IP with Chicago Sky Blue 6B ink and the macroscopic presence of their secondary lymphoid tissues determined at post-mortem 7 d later. Throughout this study the ear pinna was used as the site of ID parasite injection. The mandibular lymph nodes, sub-mandibular lymph nodes and superficial parotid lymph nodes are considered to drain this anatomical region. As anticipated, these lymph nodes were absent in LTβ^−/−^ mice (Table 1) as stimulation through the LTβR during embryonic development is essential for their formation (12, 17, 18). However, the LTβ^−/−^ mice retained the spleen (Table 1).

**Table 1:** Incidence of lymphoid tissues in WT and LTβ^−/−^ mice.

	**Mouse strain**							
**Lymphoid tissue**	**WT**				**LTβ^−/−^**			
	**M1**	**M2**	**M3**	**M4**	**M1**	**M2**	**M3**	**M4**
Mandibular lymph node	2	2	2	2	0	0	0	0
Sub-mandibular lymph node	2	2	2	2	0	0	0	0
Superficial parotid lymph node	2	2	2	2	1	0	0	0
Spleen	1	1	1	1	0	0	0	0

*Number of secondary lymphoid tissues detected macroscopically in wild-type (WT) and LTβ^−/−^ mice. Each column represents an individual mouse (M), 4 mice/group*.

### Increased Susceptibility of LTβ^-/-^ Mice to Intradermal *T. brucei* Infection

**Figure 1:**
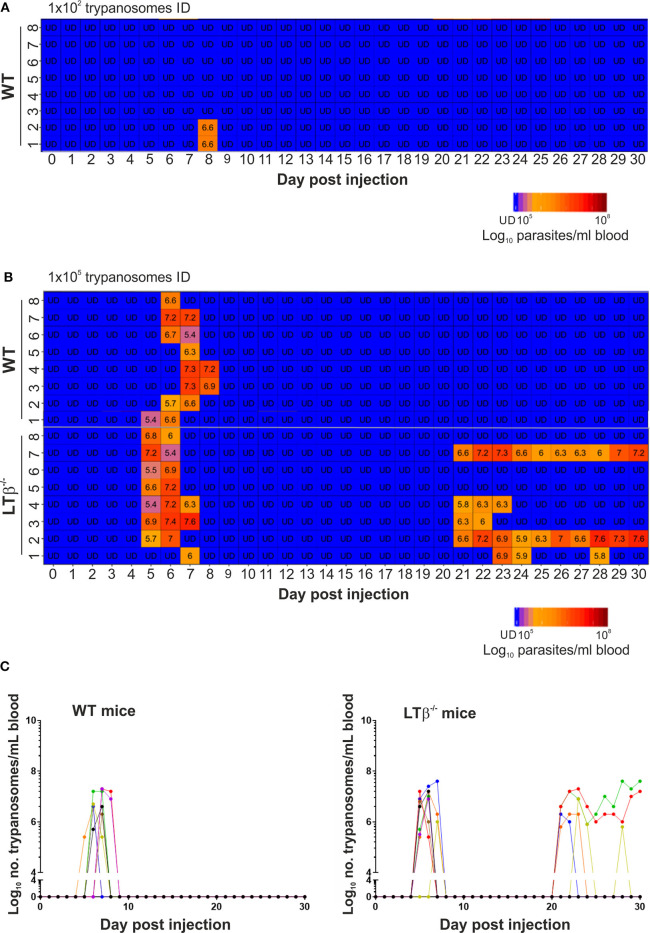
Enhanced susceptibility of LTβ^−/−^ mice to ID infection with *T. brucei*. **(A)** Wild-type (WT) mice (*n* = 8) were injected ID with a 1 × 10^2^ dose of *T. brucei* STIB 247 parasites and blood parasitemia levels determined at daily intervals. Heatmap shows the blood parasitemia (log_10_ number of trypanosomes/ml of blood) in each mouse. Each row represents data from an individual mouse. UD = below the limit of detection, ~5.4 log_10_ parasites/ml. **(B)** Groups of WT and LTβ^−/−^ mice (*n* = 8 mice/group) were injected ID with a 1 × 10^5^ dose of *T. brucei* STIB 247 parasites and blood parasitemia levels determined at daily intervals. Heatmap shows the blood parasitemia (log_10_ number of trypanosomes/ml of blood) in each mouse. Each row represents data from an individual mouse. UD, below the limit of detection, ~5.4 log_10_ parasites/ml. **(C)** Charts show parasitemia profiles (log_10_ number of trypanosomes/ml of blood) in each mouse from each group following ID injection with 1 × 10^5^ dose of T. brucei STIB 247 parasites. Each line represents data from an individual mouse.

Independent studies using the *T. brucei* strain 10–26 have estimated that the ID route of infection is ~100X less efficient than the IP route (6). Although infected tsetse flies are likely to transmit low numbers of trypanosomes during blood feeding, ID injection of WT mice with a 1 × 10^2^ dose of *T. brucei* strain 10–26 parasites was insufficient to establish infection (6). Here, WT control mice (*n* = 8/group) were also injected ID into the ear pinna with 1 × 10^2^ *T. brucei* STIB 247 parasites, and blood parasitemias assessed daily for 30 d using the rapid matching method (19). As this assay has a detection threshold of ~4 × 10^5^ parasites/ml blood, parasitemias below this level were classified as below the limit of detection. Consistent with the above study, our data show that when WT mice were injected ID with 1 × 10^2^ trypanosomes, only two of eight mice had a detectable parasitemia for short duration during the 30 d observation period (Figure 1A). Therefore, since mice are considered to be at least 100X less susceptible to ID *T. brucei* infection, in the subsequent experiments below the mice were injected ID with 1 × 10^5^ trypanosomes to ensure that all the recipient WT mice developed a detectable parasitemia.

Next, groups of LTβ^−/−^ mice and WT control mice (*n* = 8/group) were injected ID with 1 × 10^5^ *T. brucei* STIB 247 parasites, and blood parasitemias assessed daily for 30 d. After infection with this dose of parasites all the mice developed a detectable parasitemia. Furthermore, the onset and duration of the first detectable parasitemia wave in the bloodstream of each mouse group was similar (Figures 1B,C): WT mice, 6–8 d post-infection (dpi); LTβ^−/−^ mice, 5–7 dpi. Furthermore, the mean parasite burdens at the peak of the first parasitemia wave were also similar in each mouse group (Figures 1B,C): WT mice, 8 × 10^6^/ml parasites/ml; LTβ^−/−^ mice, 1 × 10^7^ parasites/ml; *P* = 0.460, Student's *t*-test, *n* = 8/group.

None of the ID-injected WT mice displayed any relapse in their parasitemias during the remainder of the 30 d observation period. In contrast, five of eight of the LTβ^−/−^ mice relapsed between 21 and 30 dpi, displaying subsequent parasitemia waves (Figures 1B,C). These data clearly show that ID injected *T. brucei* can successfully establish infection in the bloodstream of LTβ^−/−^ mice despite the absence of the draining lymph nodes. However, the LTβ^−/−^ mice have a reduced ability to control an ID *T. brucei* infection when compared to WT mice.

### LTβ^-/-^ Mice Have Disturbed Splenic Microarchitecture and Reduced Serum Class-Switched Immunoglobulin Levels Following Trypanosome Infection

**Figure 2:**
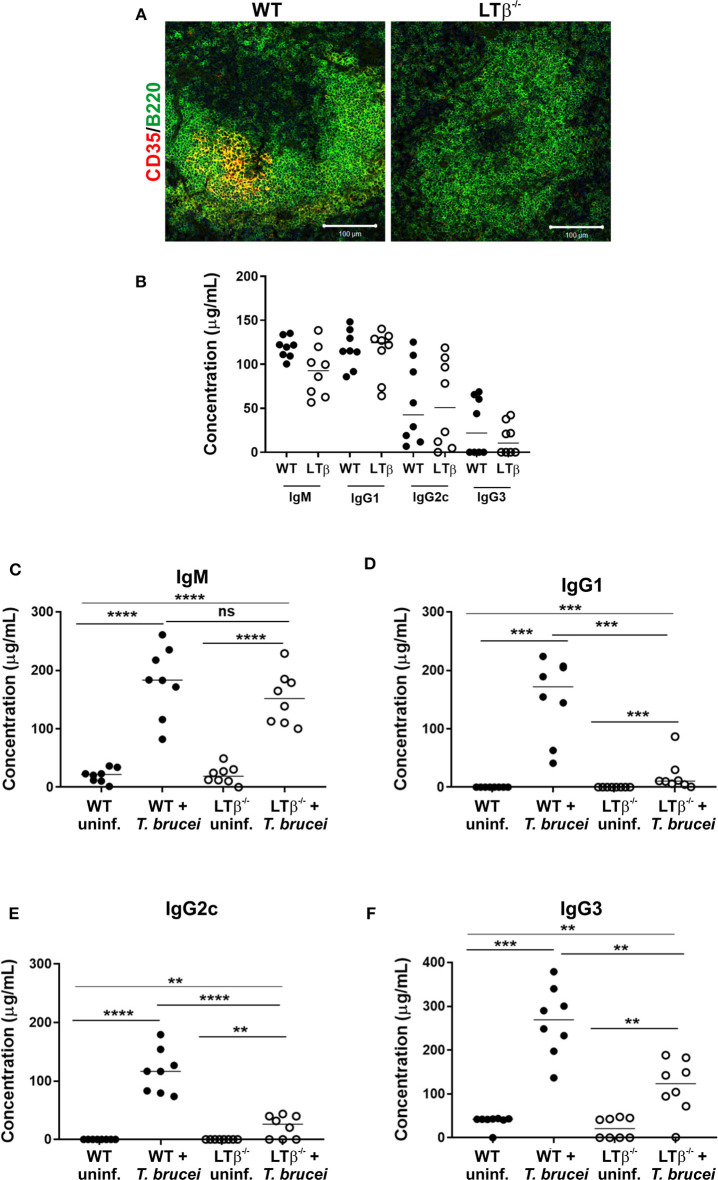
LTβ^−/−^ mice have disturbed splenic microarchitecture and reduced serum class-switched immunoglobulin levels after trypanosome infection. **(A)** Spleen sections from wild-type (WT) and LTβ^−/−^ mice were immunostained to detect follicular dendritic cells (FDC, CD35+ cells, red) and B cells (B220+ cells, green). The spleens of LTβ^−/−^ mice lacked FDC networks and had disturbed B cell follicles. **(B)** Total serum IgM, IgG1, IgG2c, and IgG3 concentrations in uninfected naïve WT and LTβ^−/−^ mice determined by ELISA (*n* = 8 mice/group). **(C–F)** Groups of WT and LTβ^−/−^ mice (*n* = 8 mice/group) were injected ID with a 1 × 10^5^ dose of *T. brucei* STIB 247 parasites and serum concentrations of trypanosome-specific **(C)** IgM, **(D)** IgG1, **(E)** IgG2c and **(F)** IgG3 determined by ELISA. Uninf., uninfected. Closed circles, WT mice; open circles, LTβ^−/−^ mice. Each point represents data from an individual mouse. Horizontal bar, median. Statistical analyses: **(B)** Student's *t*-test; **(C)** Student's *t*-test; **(D)** Mann-Whitney *U*-test; **(E)** Student's *t*-test; **(F)** Mann-Whitney *U*-test; **P < 0.01; ***P < 0.001; ****P < 0.0001.

Constitutive post-natal LTβR-stimulation is also important for the maintenance of organized B cell follicles and the stromal follicular dendritic cells (FDC) within them (20). As a consequence, the spleens of LTβ^−/−^ mice lacked CD35-expressing FDC and had disturbed B cell follicles that presented as ring-like structures (Figure 2A).

The retention of antigens on the surface of FDC is essential for GC formation and the production of high-affinity antigen-specific immunoglobulin (Ig) isotype class-switched antibodies by B cells (21). The levels of total IgM, IgG1, IgG2c, and IgG3 antibodies in the serum of uninfected naïve WT and LTβ^−/−^ mice were similar (Figure 2B), consistent with previous data (10). By 30 d following ID infection with *T. brucei*, the sera of infected mice from each group contained significantly elevated levels of trypanosome-specific IgM (Figure 2C). However, whereas elevated levels of trypanosome-specific class-switched IgG1, IgG2c and IgG3 antibodies were detected in the sera of WT mice, significantly lower levels, if any, were detected in the sera of LTβ^−/−^ mice (Figures 2D–F).

### WT Bone Marrow Restores Splenic Microarchitecture in LTβ^-/-^ Mice and Reduces Their Susceptibility to ID *T. brucei* Infection

**Figure 3:**
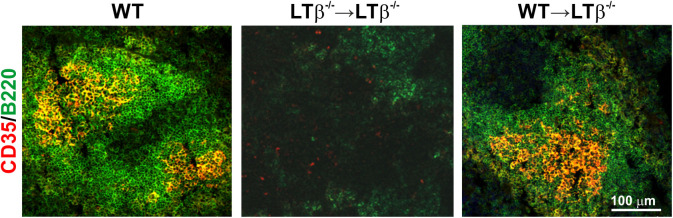
Reconstitution of LTβ^−/−^ mice with wild-type (WT) bone marrow restores the disturbed splenic microarchitecture. LTβ^−/−^ mice were γ-irradiated and reconstituted with donor bone marrow from WT mice (WT → LTβ^−/−^ mice) or LTβ^−/−^ mice (LTβ^−/−^ → LTβ^−/−^ mice). Ten weeks later spleens were collected and sections immunostained to detect follicular dendritic cells (FDC, CD35+ cells, red) and B cells (B220+ cells, green). FDC differentiation was restored in the spleens of WT → LTβ^−/−^ mice.

We next determined whether the increased susceptibility of LTβ^−/−^ mice to ID *T. brucei* infection was a consequence of their impaired ability to produce trypanosome-specific class-switched Ig isotypes. Constitutive LTβR-mediated stimulation via lymphocyte-derived LTα_1_β_2_ is essential for the maintenance of FDC networks and lymphoid tissue microarchitecture (18, 20, 22). As mature FDC are important for the efficient production of antigen-specific class-switched antibodies by B cells (21, 23), FDC differentiation was induced in the remaining lymphoid tissues of LTβ^−/−^ mice. Thus, the reconstitution of LTβ^−/−^ mice with LT-expressing donor bone marrow from WT mice (termed WT → LTβ^−/−^ mice, hereafter) stimulated the differentiation of FDC from stromal-derived precursor cells in the spleens of the recipient LTβ^−/−^ mice (Figure 3). However, this treatment does not induce the development of the missing secondary lymphoid tissues (24). Conversely, when LTβ^−/−^ mice were reconstituted with LTβ^−/−^ donor bone marrow as a negative control (termed LTβ^−/−^ → LTβ^−/−^ mice, here after), no induction of FDC network formation was observed (Figure 3).

**Figure 4:**
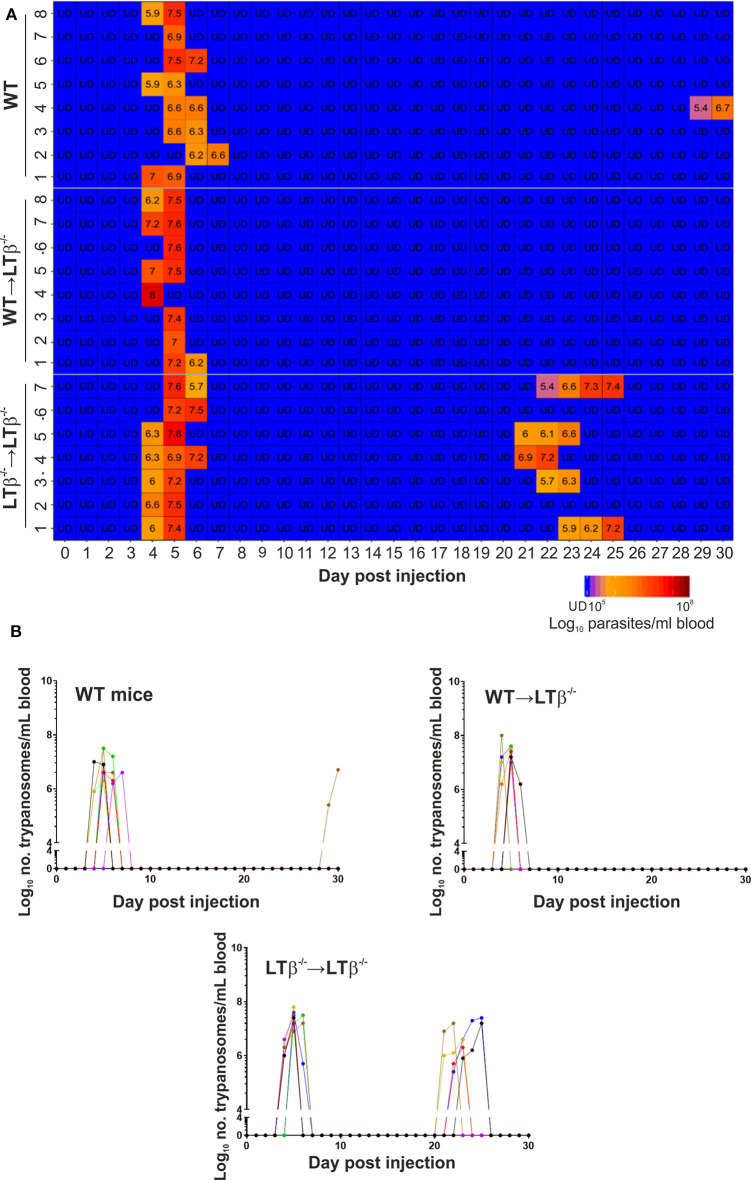
Enhanced susceptibility of LTβ^−/−^ mice to ID infection with *T. brucei*. Groups of LTβ^−/−^ mice were γ-irradiated and reconstituted with donor bone marrow from wild-type (WT) mice (WT → LTβ^−/−^ mice) or LTβ^−/−^ mice (LTβ^−/−^ → LTβ^−/−^ mice). Ten weeks later groups of WT mice, WT → LTβ^−/−^ mice and LTβ^−/−^ → LTβ^−/−^ mice (*n* = 7–8 mice/group) were injected ID with a 1 × 10^5^ dose of *T. brucei* STIB 247 parasites and blood parasitemia levels determined at daily intervals. **(A)** Heatmap shows the blood parasitemia (log_10_ number of trypanosomes/ml of blood) in each mouse. Each row represents data from an individual mouse. UD, below the limit of detection, ~5.4 log_10_ parasites/ml. **(B)** Charts show parasitemia profiles (log_10_ number of trypanosomes/ml of blood) in each mouse from each group following ID injection with 1 × 10^5^ dose of *T. brucei* STIB 247 parasites. Each line represents data from an individual mouse.

Next, groups of WT → LTβ^−/−^ mice, LTβ^−/−^ → LTβ^−/−^ mice and un-irradiated WT control mice were ID injected with 1x10^5^ *T. brucei* STIB 247 parasites and blood parasitemias assessed daily for 30 d. The onset and duration of the first detectable parasitemia waves in the bloodstream of each mouse group were similar (Figures 4A,B). However, noticeable differences in the parasitemia kinetics were evident during the later stages of the infections. The trypanosome infection relapsed in most of the LTβ^−/−^ → LTβ^−/−^ mice from ~21 dpi with the detection of subsequent parasitemia waves (Figures 4A,B). These data show that LTβ^−/−^ → LTβ^−/−^ mice, like LTβ^−/−^ mice (Figure 1), have increased susceptibility to ID *T. brucei* infection. In contrast, none of the WT → LTβ^−/−^ mice, and only one of eight of the ID injected un-irradiated WT mice, had any detectable relapses in their parasitemias during the remainder of the experiment (Figures 4A,B).

### Reconstitution of LTβ^-/-^ Mice With WT Bone Marrow Induces Their Ability to Produce Ig Isotype Class-Switched Antibodies

**Figure 5:**
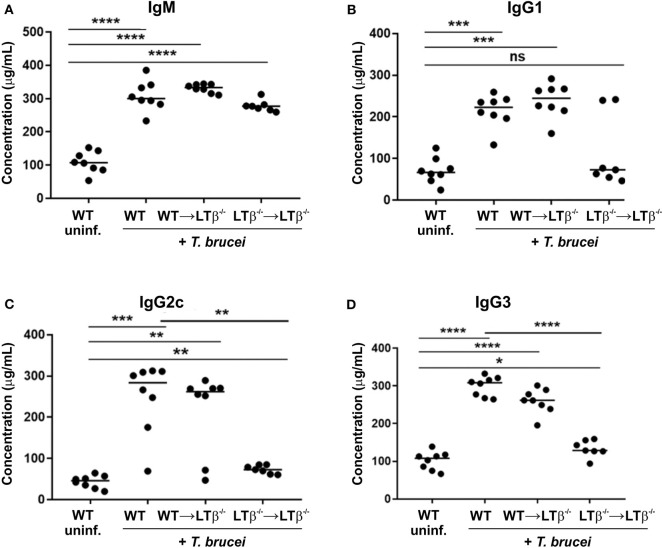
Reconstitution of LTβ^−/−^ mice with wild-type (WT) bone marrow restores their ability to produce Ig isotype class-switched antibodies. Groups of LTβ^−/−^ mice were γ-irradiated and reconstituted with donor bone marrow from WT mice (WT → LTβ^−/−^ mice) or LTβ^−/−^ mice (LTβ^−/−^ → LTβ^−/−^ mice). Ten weeks later groups of WT mice, WT → LTβ^−/−^ mice and LTβ^−/−^ → LTβ^−/−^ mice (*n* = 7–8 mice/group) were injected ID with 1x10^5^ dose of *T. brucei* STIB 247 parasites and 30 d later concentrations of total serum **(A)** IgM, **(B)** IgG1, **(C)** IgG2c, and **(D)** IgG3 determined by ELISA. Uninf., uninfected. Each point represents data from an individual mouse. Horizontal bar, median. Statistical analyses: **(A)** Student's *t*-test; **(B)** Mann-Whitney *U*-test; **(C)** Mann-Whitney U test; **(D)** Student's *t*-test; *P < 0.05; **P < 0.01; ***P < 0.001; ****P < 0.0001.

We next determined whether the reconstitution of LTβ^−/−^ mice with WT bone marrow induced the ability to produce class-switched Ig isotypes. By 30 d following ID injection with *T. brucei*, similarly elevated levels of total IgM were detected in the sera of WT mice, WT → LTβ^−/−^ mice and LTβ^−/−^ → LTβ^−/−^ mice (Figure 5A). However, the sera of WT → LTβ^−/−^ mice contained significantly elevated levels of total class-switched IgG isotypes when compared to uninfected WT controls (Figures 5B–D). These data suggest that the restoration of splenic microarchitecture in WT → LTβ^−/−^ mice coincided with the ability to produce class-switched Ig isotypes.

**Figure 6:**
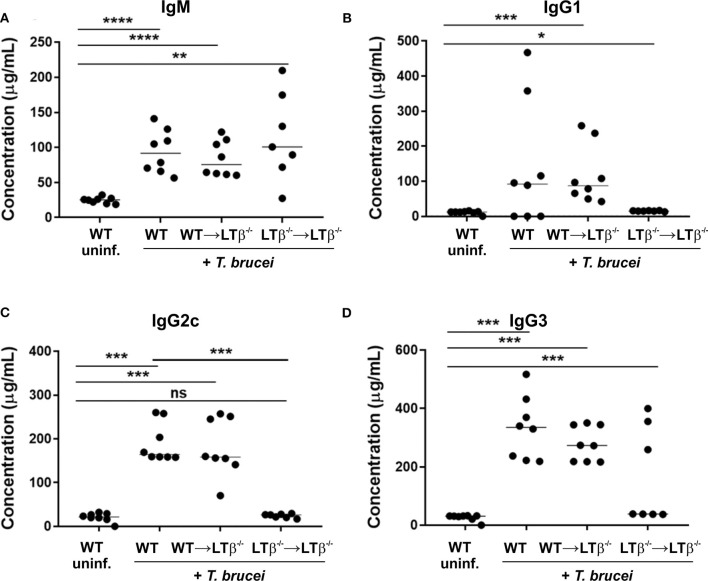
Reconstitution of LTβ^−/−^ mice with wild-type (WT) bone marrow restores their ability to produce trypanosome-specific Ig isotype class-switched antibodies. Groups of LTβ^−/−^ mice were γ-irradiated and reconstituted with donor bone marrow from WT mice (WT → LTβ^−/−^ mice) or LTβ^−/−^ mice (LTβ^−/−^ → LTβ^−/−^ mice). Ten weeks later groups of WT mice, WT → LTβ^−/−^ mice and LTβ^−/−^ → LTβ^−/−^ mice (*n* = 7–8 mice/group) were injected ID with a 1 × 10^5^ dose of *T. brucei* STIB 247 parasites and 30 d later concentrations of total serum **(A)** IgM, **(B)** IgG1, **(C)** IgG2c, and **(D)** IgG3 determined by ELISA. Uninf., uninfected. Each point represents data from an individual mouse. Horizontal bar, median. Statistical analyses: **(A)** Student's *t*-test; **(B)** Mann-Whitney *U* test; **(C)** Mann-Whitney *U* test; **(D)** Student's *t*-test; *P < 0.05; **P < 0.01; ***P < 0.001; ****P < 0.0001.

Finally, we compared the levels of trypanosome-specific antibodies in the sera of infected WT mice, WT → LTβ^−/−^ mice and LTβ^−/−^ → LTβ^−/−^ mice. Sera from infected mice from each group contained significantly elevated levels of trypanosome-specific IgM (Figure 6A). However, whereas WT mice and WT → LTβ^−/−^ mice produced similarly elevated levels of trypanosome-specific class-switched IgG1, IgG2c, and IgG3 antibodies, much lower levels if any were detected in the sera of LTβ^−/−^ → LTβ^−/−^ mice (Figures 6B,C). Thus, these data suggest that the restoration of the microarchitecture in the spleens of WT → LTβ^−/−^ mice enabled the production of trypanosome-specific class-switched antibodies.

## Discussion

Following ID injection into the host's skin the extracellular trypanosomes invade the afferent lymphatics (1) from where they infect the draining lymph nodes and begin to disseminate systemically (4, 5). For example, during the initial period following ID infection of cattle and goats with *T. vivax* by tsetse fly bite, the parasites were first encountered in the draining pre-scapular lymph nodes before the bloodstream (2, 3). Similar disease kinetics have also been reported in mice infected with *T. brucei* by tsetse fly bite (5). Circulating lymphocytes/leukocytes, antigens and other components within the lymph fluid can enter the bloodstream via the right lymphatic duct on the right side of the body, or the thoracic duct on the left side of the body. From these lymphatic ducts, the passage of lymph into the blood circulatory system is achieved either via the right or left subclavian vein. We show that the ability of the ID injected trypanosomes to establish systemic infection was not compromised in mice lacking the draining lymph nodes. Our data therefore suggest that following their initial invasion into the host's lymphatics, the trypanosomes can directly enter the bloodstream via the lymphatic ducts and disseminate further without the requirement for prior adaptation or amplification within the draining lymph nodes.

Although the LTβ^−/−^ mice lacked most peripheral lymph nodes, had disorganized microarchitecture within their spleens and lacked the ability to mount trypanosome-specific class-switched IgG responses, the magnitude and duration of the initial parasitemia wave was similar when compared to WT mice. The increased ability of C57BL/6 mice to control the first parasitemia wave when compared to C3H/He mice, has been suggested to be associated with their relative ability to produce trypanosome-specific Ig (25). During the first few days after trypanosome infection the initial and predominant parasite-specific antibodies that are produced by the host are of the IgM isotype that recognize parasite surface antigens (26–29). The production of parasite-specific IgM was shown to be important for control of *T. b. evansi* infection (30). In contrast, a study using IgM-deficient mice has suggested only a limited role for IgM in controlling infection with clonal pleomorphic *T. brucei* AnTat 1.1E parasites (31). However, the compensatory effects of other Ig isotypes in host protection could not be entirely excluded. Macrophages have also been suggested to play an important role in the innate immune system's ability to control the infection by phagocytosing and destroying the parasites in tissues (32). For example, the Kupffer cells in the liver can aid the elimination of *T. congolense* from the bloodstream through IgM- and IgG-dependent phagocytosis of the parasites (33). In the current study the levels of parasite-specific IgM detected in the sera of LTβ^−/−^ mice were similar to those in WT controls. This implies that a combination of parasite-specific IgM and the responses and actions of innate phagocytes are likely to play an important role in controlling the initial phase of the infection to the infecting parasite clones via antibody-dependent phagocytosis. It remains to be determined whether B1 B-cell subsets or B2 B-cell subsets, including GC B-cell, follicular B-cell and marginal zone (MZ) B cells, are the sources of the parasite-specific IgM in the current study. However, since mice that lack LTβR-signaling also lack GC, B cell follicles and MZ in their spleens (34, 35), B2 B cells are unlikely to be a major source of trypanosome-specific IgM in the ID infected LTβ^−/−^ mice. An independent study has also suggested that MZ B cells are depleted following infection with trypanosomes by the IP route (36). The requirement for GC B cells is less clear. Whereas, data suggest that GC are not induced in the spleen during trypanosome infections (37), GC B cells have been reported to accumulate in this tissue during the later stages of IP infection with pleomorphic *T. brucei* AnTat 1.1E parasites (36). In contrast, GC formation has been described in the spleens of mice after IP infection with *T. congolense* (38). Whether GC are also induced in the skin draining lymph nodes during *T. brucei* infection is not known.

Closer analysis suggested that the onset of the first parasitemia wave in the ID infected LTβ^−/−^ mice typically occurred a day earlier than ID infected WT mice. The precise reason for this earlier onset is uncertain, but it is plausible that after ID infection in WT mice some of the parasites are initially retained in, or migration is slowed by, the local draining lymph node. Alternatively, some parasites may also be phagocytosed and destroyed by resident macrophages as they travel through the draining lymph node. Since LTβ^−/−^ mice lacked the draining lymph nodes it is possible that these effects were avoided, enabling a higher burden of parasites to initially enter the blood-stream, leading to the slightly earlier development of the first parasitemia peak.

As the infection progressed the LTβ^−/−^ mice were unable to successfully control the parasitemia when compared to WT mice. Following the initial parasitemia wave the production of trypanosome-specific Ig isotype class-switched IgG antibodies is essential for the control of the subsequent relapses in the parasitemia (29, 31, 39, 40). The parasite class-switched IgG antibodies that are induced during this phase of the disease target the trypanosome's variable surface glycoprotein coat with high affinity (40, 41). Furthermore, differences in the magnitude of the host's parasite-specific class-switched IgG response can have a direct influence on the pathogenesis of *T. congolense* infection in mice (42) and cattle (43). For example, relatively resistant C57BL/6 mice produce high levels of trypanosome-specific IgG isotypes when compared to relatively susceptible A/J mice (42).

Here, the increased susceptibility of LTβ^−/−^ mice to ID *T. brucei* infection coincided with significantly reduced serum levels of parasite-specific class-switched Ig isotypes. Since stimulation through the LTβR is essential for the organization of the B cell follicles within the secondary lymphoid tissues, LTβ^−/−^ mice have disorganized B cell follicles (9, 12) and a significantly reduced ability to elicit high affinity antigen-specific class-switched IgG responses (35). Consistent with these studies the ID infected LTβ^−/−^ mice also had significantly reduced serum levels of trypanosome-specific IgG1, IgG2c, and IgG3. This implies that the reduced ability of the LTβ^−/−^ mice to effectively control the parasitemia during the later stages of the infection was at least in part a consequence of their reduced ability to produce parasite-specific class-switched IgG antibodies. To test this hypothesis the splenic microarchitecture and the ability of LTβ^−/−^ mice to produce antigen-specific Ig class-switched antibodies was restored by reconstitution with bone marrow from WT donor mice. Our data clearly show that the WT → LTβ^−/−^ mice produced significant levels of trypanosome-specific class-switched IgG1, IgG2c and IgG3, and this coincided with the ability of the WT → LTβ^−/−^ mice to control further relapses in the parasitemia to a similar extent as WT mice. Future studies using transgenic mice with specific deficiencies in IgG1 (44) or IgG3 (45) will help resolve the individual roles of parasite-specific class-switched antibody isotypes in the control of ID *T. brucei* infections.

Our studies using LTβ^−/−^ mice suggest that the presence of the draining lymph nodes has little, if any, impact on susceptibility to ID *T. brucei* infection. However, our data indicate that the status of the microarchitecture of the spleen has an important role in controlling the trypanosome infection. In the current study the disorganized microarchitecture in the spleens of LTβ^−/−^ mice and LTβ^−/−^ → LTβ^−/−^ mice coincided with increased susceptibility to ID *T. brucei* infections. Conversely, the organized splenic microarchitecture in WT mice and WT → LTβ^−/−^ mice coincided with their increased ability to control subsequent parasitemia waves. These data are consistent with data in an independent study, which showed that in the absence of the spleen, parasite-specific IgG2a/c and IgG3 responses were impaired and this coincided with increased susceptibility to *T. congolense* infection (40). Our data are also consistent with those in a study using mice lacking the B cell adaptor molecule Bam32 (38). When compared to WT controls, these mice also had increased disease severity, which correlated with impaired splenic GC formation and a diminished ability to produce parasite-specific IgG after IP *T. congolense* infection. Others, however, have suggested that the induction of anti-trypanosome antibody responses can occur independently of the lymph nodes and spleen, during experimental IP infection with *T. brucei* AnTat 1.1E parasites (46). Furthermore, another study suggested that LTβ^−/−^ mice had reduced susceptibility to IP infection with *T. congolense* (47). The reason for the discrepancy between these studies and our own is uncertain, but may be related to differences in route of infection, parasite burden and parasite strains. When LTα^−/−^ mice (as used by Magez and colleagues, 2002, (46) are immunized with low doses of T-cell-dependent antigens their ability to produce high affinity antigen-specific IgG1 is reduced. Conversely, when these mice are immunized with high doses of antigens they produce similar levels of high affinity antigen-specific IgG1 to WT mice (13, 34). Studies in mice also show that the route of trypanosome injection can have a significant influence on disease susceptibility and pathogenesis, with ID infections with *T. brucei* or *T. congolense* being 100X less efficient than IP infections (6). Using the *T. brucei* strain 10-26, Wei and colleagues showed that a detectable parasitemia was established in all mice injected with as few as 10^2^ parasites by the IP route. However, the same dose was insufficient to establish a detectable infection by the ID route. Indeed, a detectable parasitemia was only observed in all of the recipient mice when injected ID with 10^4^ parasites. Wei and colleagues also showed that when the mice were infected by the IP route the trypanosomes established a detectable parasitemia earlier than after ID injection, with a higher parasite burden (6). In the studies by Magez et al. (46) and Okwor et al. (47) the mice were similarly injected with trypanosomes by the IP route. Thus, it is plausible that the IP infections led to a significantly higher and earlier antigen burden in the LTα^−/−^ mice enabling high affinity parasite-specific IgG responses to be induced despite the absence of organized splenic microarchitecture (13, 34). To resolve these issues additional studies are now required to definitively address the contributions of the skin draining lymph nodes and the spleen for the induction and maintenance of parasite-specific antibody responses after ID trypanosome infection. This could include comparison of parasitemia kinetics, disease susceptibility and parasite-specific B cell responses in intact and splenectomised WT mice and LTβ^−/−^ mice.

When considered in the context of similar studies, our data imply that the first wave of the parasitemia after ID infection with *T. brucei* is predominantly controlled by innate mechanisms, most likely including non-class switched IgM and the engulfment of the parasites by phagocytes. However, control of the subsequent parasitemia waves requires organized B cell follicles in the spleen and the production of trypanosome-specific class-switched IgG. Drug resistance, as well as adverse side-effects, continue to significantly impact on the ability to successfully treat and control trypanosome infections. A more thorough understanding of the host factors that are essential for the efficient control of ID trypanosome infections could identify novel therapeutics and aid the development of protective vaccines.

## Data Availability Statement

All datasets generated for this study are included in the article/supplementary material.

## Ethics Statement

The animal study was reviewed and approved by The Roslin Institute's and University of Edinburgh's ethics committees.

## Author Contributions

LM and NM conceived the study and obtained funding. OA, LM, and NM designed the study. OA, EP, and BB performed the experiments and acquired these data. All authors interpreted these data and contributed to the final version of the manuscript.

## Conflict of Interest

The authors declare that the research was conducted in the absence of any commercial or financial relationships that could be construed as a potential conflict of interest.
